# Glutathione synthesis is compromised in erythrocytes from individuals with HIV

**DOI:** 10.3389/fphar.2014.00073

**Published:** 2014-04-11

**Authors:** Devin Morris, Judy Ly, Po-Ting Chi, John Daliva, Truongson Nguyen, Charleen Soofer, Yung C. Chen, Minette Lagman, Vishwanath Venketaraman

**Affiliations:** ^1^Department of Basic Medical Sciences, College of Osteopathic Medicine of the Pacific, Western University of Health SciencesPomona, CA, USA; ^2^Graduate College of Biomedical Sciences, Western University of Health SciencesPomona, CA, USA

**Keywords:** glutathione, HIV, GSS, GCL, GSR

## Abstract

We demonstrated that the levels of enzymes responsible for the synthesis of glutathione (GSH) such as glutathione synthase (GSS), glutamate-cysteine ligase-catalytic subunit (GCLC), and glutathione reductase (GSR) were significantly reduced in the red blood cells (RBCs) isolated from individuals with human immunodeficiency virus (HIV) infection and this reduction correlated with decreased levels of intracellular GSH. GSH content in RBCs can be used as a marker for increased overall oxidative stress and immune dysfunctions caused by HIV infection. Our data supports our hypothesis that compromised levels of GSH in HIV infected individuals’ is due to decreased levels of GSH-synthetic enzymes. The role of GSH in combating oxidative stress and improving the functions of immune cells in HIV patients’ indicates the benefit of an antioxidant supplement which can reduce the cellular damage and promote the functions of immune cells.

## INTRODUCTION

Roughly 34 million people around the world are infected with human immunodeficiency virus (HIV). Since its first reporting in 1981, the beginning of an epidemic, 60 million people have contracted HIV and an estimated 30 million have died due to HIV related causes (World Health Organization, 2010). HIV infection is associated with a wide range of different opportunistic infections that are usually the prime suspect for patients’ poor survival. Among the array of opportunistic infections, one of the leading life-threatening infection common among HIV positive individuals with compromised immune system is *Mycobacterium tuberculosis*. Especially in developing countries, as many as eighty percent of people with AIDS are at risk of developing tuberculosis (TB) (World Health Organization, 2009). HIV’s primary targets *in vivo* are blood monocytes, CD4 T lymphocytes, and resident macrophages. Due to HIV’s high affinity for infecting and killing CD4+ T lymphocytes, cell-mediated immunity is drastically lowered. This results in greater probability for opportunistic infections, primarily *M. tuberculosis* (Levy, 1993; Pantaleo et al., 1993; Droge and Holm, 1997; Herzenberg et al., 1997).

Glutathione (GSH) is a major component involved in the control and maintenance of cellular redox state and cellular homeostasis (Griffith, 1999). In addition, GSH is also important in an array of cellular functions such as protein synthesis, transport across membranes, receptor action, and cell growth (Griffith, 1999). As a natural antioxidant, GSH scavenges peroxide species. Low levels of GSH have been shown to play a role in the apoptosis of CD4+ T cells, which is the major pathology of the HIV infection, therefore signifying the importance of GSH (Levy, 1993; Pantaleo et al., 1993; Droge and Holm, 1997; Herzenberg et al., 1997).

Glutathione is produced by almost all cell types and are present in two forms, reduced (*r*GSH) and oxidized (GSSG). *r*GSH is synthesized by two different mechanisms. *De novo* synthesis of *r*GSH occurs in a two-step process mediated by two different enzymes, glutamate-cysteine ligase (GCL) and glutathione synthase (GSS). *r*GSH is also synthesized via the reduction of GSSG by glutathione reductase (GSR; Staal, 1998). In this study, we went beyond the innate immune response components and investigated the changes in the levels of GSH in red blood cells (RBCs) isolated from individuals with HIV infection. We hypothesized that compromised levels of GSH in HIV-infected individuals is due to decreased levels of enzymes that are involved in the synthesis of GSH. Since RBCs are systemically present in abundance, we tested our hypothesis by determining the extent to which the levels of GSH-synthetic enzymes are compromised in RBCs derived from individuals with HIV infection and correlating decreased levels of GSH-synthetic enzymes with deficiency in the levels of GSH.

## MATERIALS AND METHODS

### SUBJECTS

The protocol was approved by Institutional Review Board with the requirement that each volunteer recruited would need to be given a consent form that described the basis and the procedures of the study. A signed informed consent from each volunteer that agreed to participate was obtained. A total of 16 volunteers (eight healthy subjects and eight individuals with HIV infection) were recruited for the study. Individuals with HIV infection were recruited from the Foothills AIDS project. Healthy subjects without HIV infection or a history of TB were recruited from the staff of Western University of Health Sciences. All HIV-infected volunteers had been diagnosed with HIV-1, were taking some form of anti-retroviral treatment, and had CD4+ T-cell counts between 271 and 1415 cells per mm^3^. Thirty five milliliters (mL) of blood was drawn once from both healthy volunteers and individuals with HIV infection.

### ERYTHROCYTE ISOLATION

Red blood cells were isolated from whole blood by density gradient centrifugation with FICOLL-Paque (GE Healthcare, 17-440-02). RBCs that aggregated as the bottom layer were collected and stored at -20°C in a cell lysis/protein storage buffer [20 mM Tris, 100 mM NaCl (Amresco, N653), 1X protease inhibitor cocktail (Amresco, M221)] for western blot analysis.

### GEL ELECTROPHORESIS AND WESTERN BLOT ANALYSIS IN RBC LYSATES FROM HEALTHY AND HIV^+^ SUBJECTS

Total protein content was determined using Coomassie blue colorimetric assay (Thermo Scientific, PI-23200). 200 μg of total RBC proteins per sample were separated via denaturing polyacrylamide electrophoresis (12%). Separated proteins were transferred to a Polyvinylidene fluoride membrane (GE, PV4HY00010) by electroblotting. Membranes were blocked for 1 h at room temperature in tris buffered saline with tween 20 (TBST) and 5% non-fat dry milk followed by three washes (15 min for each wash) in TBST with mild shaking. The membranes were then incubated with a primary antibody overnight at 4°C in TBST with gentle shaking. The primary antibodies used were mouse anti-human GSS (1:1000, Abcam, ab5513), mouse anti-human GSR (1:500, Abcam, ab55075), and rabbit anti-human glutamate-cysteine ligase-catalytic subunit (GCLC; 1:250, Abcam, ab40929). Following overnight incubation with the primary antibodies, membranes were washed five times for 15 min in TBST with mild shaking. Washed membranes were incubated with a secondary antibody conjugated with horse radish peroxidase, anti-mouse (1:1000, Abcam, ab7064), or anti-rabbit (1:1000, Abcam, ab72465) in TBST for an hour at room temperature. Membranes were washed again five times for 15 min in TBST with mild shaking. Chemiluminescent substrate was applied to the membranes which were then exposed to an x-ray film (Genemate, F-9024) and developed in a dark room. Digital images of the immunoblots were captured using a Versadoc gel imaging system (Bio-rad, 4000 MP). Densitometric analysis of the images was performed using ImageJ, a free software program available from the National Institutes of Health (http://rsbweb.nih.gov/ij/).

### ASSAY OF GSH LEVELS IN RBCs FROM HEALTHY AND HIV^+^SUBJECTS

Glutathione concentrations were measured in RBCs isolated from healthy and HIV^+^ individuals by spectrophotometry using a colorimetric assay kit (Arbor Assays, K006-H1). RBCs were suspended in an ice cold 5% 5-sulfosalicylic acid dihydrate solution (MP Biomedicals, 160001-4924H). Supernatants collected after centrifugation were analyzed for the total GSH as per the manufacturer’s instructions. All GSH measurements were normalized with total protein concentrations.

### STATISTICAL ANALYSIS

Statistical analysis of the data was carried out using GraphPad Prism 6. The data was analyzed by comparing the means of *n* = 8 individuals (unless otherwise specified) using unpaired student’s *t*-tests. *P* ≤ 0.05 was considered statistically significant.

## RESULTS AND DISCUSSION

Glutathione is a tripeptide made of glutamine, cysteine, and glycine. In the *de novo* synthesis of GSH, glutamine is linked to cysteine by GCL to form -glutamylcysteine (Griffith, 1999). Then GSS links the dipeptide -glutamylcysteine to glycine to form the final GSH molecule (Griffith, 1999). The GSH redox system plays a major role in ridding the body of oxidative stress and restoring homeostasis (Griffith, 1999). To elicit antioxidant effects, GSH is converted to oxidized glutathione (GSSG) by glutathione peroxidase (GPx). GSSG can be converted back to GSH by GSR (Staal, 1998). It is important to note that only free GSH has antioxidant effects. On the other hand, GSSG lacks antioxidant functions and is a byproduct of the scavenging activity of GSH (Staal, 1998; Griffith, 1999). GSH/GSSG ratio should be maintained to optimize the GSH redox system. GCL, the rate-limiting enzyme of GSH synthesis, is composed of a catalytic subunit (GCLC) and a modulating subunit (GCLM). GCLC is the component that performs the amino acid linkage between glutamine and cysteine, whereas GCLM modulates the activity of GCLC (Huang et al., 1993).

**FIGURE 1:**
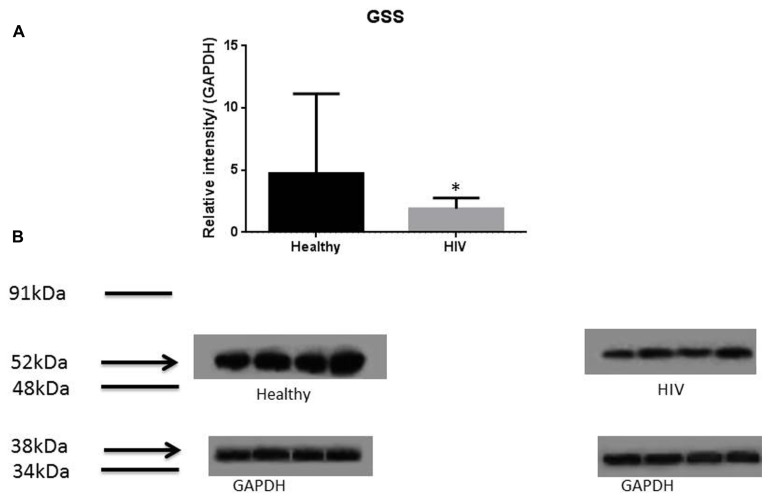
**Quantifying of GSS enzyme levels in Healthy and HIV-infected subjects.** Red blood cell samples were separated from blood of healthy volunteers and HIV-infected individuals were used for measurement of GSS, the enzyme that is involved in the second step of GSH synthesis. Electrophoresis and Western blot were used. There was a significant decrease of GSS in the RBC of HIV-infected individuals compared to healthy individuals. Data in **(A)** represents significant difference in the levels of GSS between RBCs isolated from eight healthy individuals and eight individuals with HIV infection (*p ≤ 0.05). **(B)**: Illustrates representative images of GSS and their corresponding GAPDH bands from four healthy subjects and four individuals with HIV infection.

**FIGURE 2:**
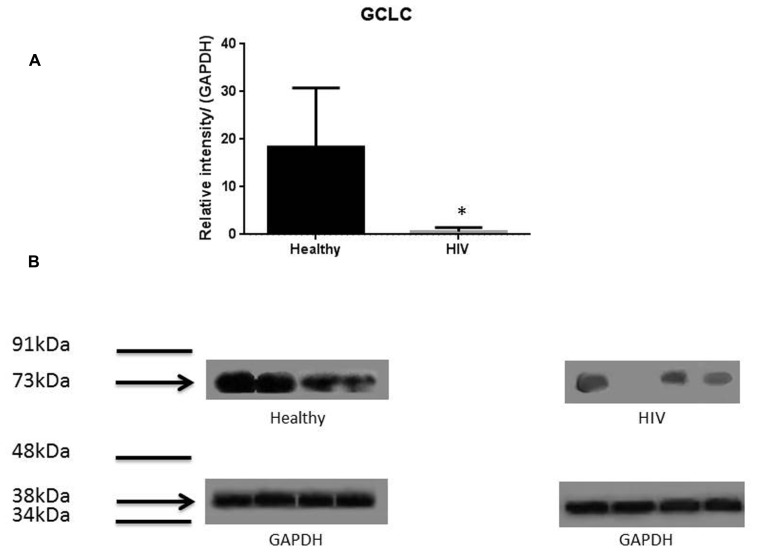
**Quantifying of GCLC enzyme levels in Healthy and HIV-infected subjects.** Red blood cell samples were separated from blood of healthy volunteers and HIV-infected individuals were used for measurement of the catalytic subunit of GCLC that is responsible for the linkage between glutamine and cysteine in the first step of GSH synthesis. Electrophoresis and Western blot were used. GCLC levels were significantly lower in the RBC of HIV-infected individuals compared to healthy subjects. Data in **(A)** represents significant difference in the levels of GCLC between RBCs isolated from eight healthy individuals and eight individuals with HIV infection (*p ≤ 0.05). **(B)**: Illustrates representative images of GCLC and their corresponding GAPDH bands from four healthy subjects and four individuals with HIV infection.

**FIGURE 3:**
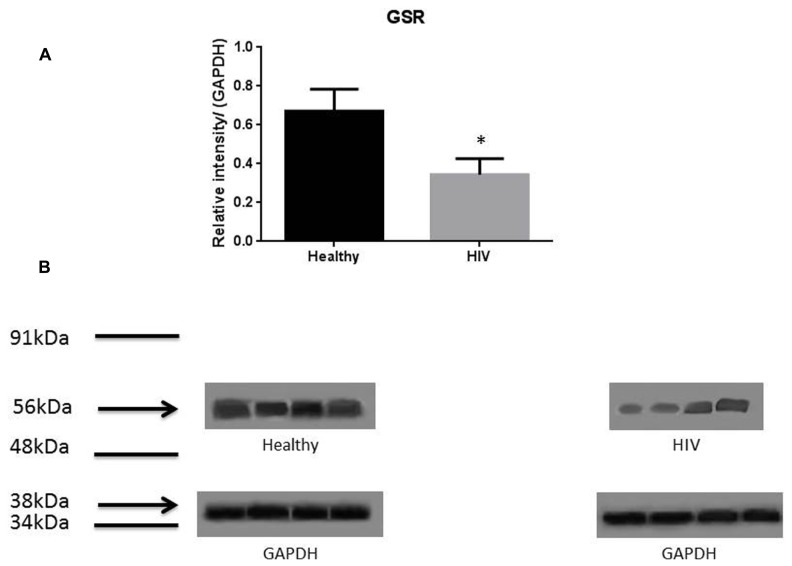
**Quantifying of GSR enzyme levels in Healthy and HIV-infected subjects.** Red blood cell samples were separated from blood of healthy volunteers and HIV-infected individuals were used for measurement of GSR. GSR converts oxidized glutathione (GSSG) to glutathione (GSH). The GSR levels in the RBC of HIV-infected individuals are lower than healthy individuals. Electrophoresis and Western blot were used. GSR levels in HIV-infected individuals are lower compared to healthy. Data in **(A)** represents significant difference in the levels of GSR between RBC isolated from five healthy individuals and five individuals with HIV infection (*p ≤ 0.05). **(B)**: Illustrates representative images of GSR and their corresponding GAPDH bands from four healthy subjects and four individuals with HIV infection.

**FIGURE 4:**
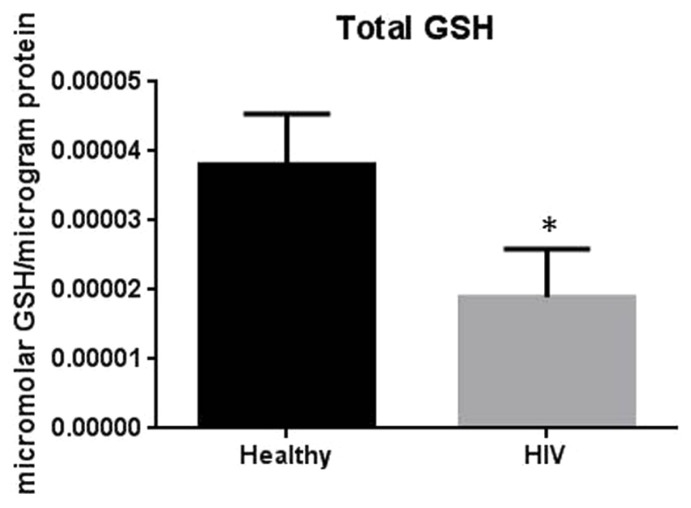
**Assay of GSH levels in RBCs from healthy subjects and individuals with HIV infection.** Analysis of total GSH which includes both free GSH and GSSG indicated significantly higher levels of GSH in healthy individuals compared to HIV positive subjects. Data in Figure 4 represents significant difference in the levels of total GSH between RBCs isolated from five healthy individuals and five individuals with HIV infection (*p=0.05).

It has previously been reported that GSH levels in the plasma, erythrocytes, and peripheral blood mononuclear cells (PBMC) of HIV^+^ individuals are compromised (Sbrana et al., 2004; Venketaraman et al., 2006; Guerra et al., 2011, 2012; Morris et al., 2012, 2013). The goal of our study is to characterize the causes for diminished levels of GSH in HIV infected individuals by determining the extent to which the levels of GCLC, GSS, and GSR are decreased in RBCs isolated from individuals with HIV infection compared to healthy subjects. Measurement of GSS and GCLC revealed a significant decrease in the levels of these enzymes present in RBCs of HIV-infected individuals compared to healthy subjects (**Figures 1** and **2**). Both GSS and GCLC are crucial enzymes that are involved in the catalytic rate limiting step and second step reaction, respectively, in the biosynthesis of GSH (Staal, 1998; Griffith, 1999; Morris et al., 2012, 2013). We also observed a significant decrease in the expression of GSR in RBCs isolated from HIV positive subjects (**Figure 3**). This explains the reason for decreased levels of GSH and the consequences related to the GSH deficiency such as loss of immune function observed in HIV patients (Venketaraman et al., 2006; Guerra et al., 2011, 2012; Morris et al., 2012, 2013). Reduced expressions of GSH synthesis enzymes in RBCs from individuals with HIV infection was accompanied by decreased levels of total GSH (**Figure 4**).

HIV^+^ individuals were also found to have increased levels of TGF-β in their plasma and macrophage supernatants (Morris et al., 2012). Moreover, TGF-β is known to block the production of GCLC which leads to decreased GSH synthesis (Morris et al., 2012). HIV-1 transactivator protein (TAT) decreases the amount of GSH present in mice through the modulation of GSH biosynthetic enzymes (Choi et al., 2000). TAT also increases free radical production. Therefore, marked increase in oxidative stress along with increased levels of TGF-β lead to the compromised levels of GSH synthesis enzymes. The master transcription factor nuclear factor (erythroid-derived 2)-like 2 (Nrf2) regulates the expression of antioxidant and phase II-metabolizing enzymes by activating the antioxidant response element (ARE) and thereby protects cells and tissues from oxidative stress. The Nrf2 gene binding to the ARE results in the upregulation of GSH synthesis enzymes such as GCLC, GCLM, and GSR. New findings argue that HIV-1-related proteins downregulate Nrf2 expression and/or activity within the alveolar epithelium, which in turn impairs antioxidant defenses and barrier function, thereby rendering the lung susceptible to oxidative stress and injury (Fan et al., 2013). Furthermore, this study suggests that activating the Nrf2/ARE pathway with the dietary supplement sulforaphane could augment antioxidant defenses and lung health inHIV-1-infected individuals (Fan et al., 2013).

We have previously reported that the virulent laboratory strain of *M. tuberculosis* H37Rv is sensitive to GSH at physiological concentrations (5 mM) when grown *in vitro* (Venketaraman et al., 2005). Thus, GSH has direct antimycobacterial activity, functioning as an effector molecule in innate defense against *M. tuberculosis* infection (Venketaraman et al., 2005; Dayaram et al., 2006). We recently reported that GSH is integral in facilitating the control of intracellular growth of *M. tuberculosis* in human macrophages (Venketaraman et al., 2005; Dayaram et al., 2006; Morris et al., 2012, 2013). These results further confirm that GSH has direct antimycobacterial activity and unfolds a novel and potentially important innate defense mechanism adopted by human macrophages to control *M. tuberculosis* infection. We also demonstrated that GSH in combination with cytokines such as IL-2 and IL-12 enhances the functional activity of natural killer (NK) cells to inhibit the growth of *M. tuberculosis* inside human monocytes (Millman et al., 2008; Guerra et al., 2012). Importantly, data from our most recent studies indicate that GSH activates the functions of T lymphocytes to control *M. tuberculosis* infection inside human monocytes (Guerra et al., 2011). These results indicate that GSH inhibits the growth of *M. tuberculosis* by both direct antimycobacterial effects as well as by activating the functions of immune cells (Venketaraman et al., 2005; Dayaram et al., 2006; Millman et al., 2008; Guerra et al., 2011, 2012; Morris et al., 2012, 2013). We also reported that the GSH concentrations were significantly lower in macrophages, NK, and T cells isolated from individuals with HIV infection compared to healthy subjects (Venketaraman et al., 2006; Guerra et al., 2011, 2012; Morris et al., 2012, 2013). Decreased levels of GSH in macrophages, NK ,and T cells derived from individuals with HIV infection was accompanied by diminished control of intracellular *M. tuberculosis* infection (Venketaraman et al., 2006; Guerra et al., 2011, 2012; Morris et al., 2012, 2013). Our group is a pioneer in reporting that GSH levels were decreased in macrophages, T cells, and NK cells from individuals with HIV infection and correlating decreased GSH levels with impaired innate and adaptive immune responses against *M. tuberculosis* infection (Venketaraman et al., 2006; Guerra et al., 2011, 2012; Morris et al., 2012, 2013).

**FIGURE 5:**
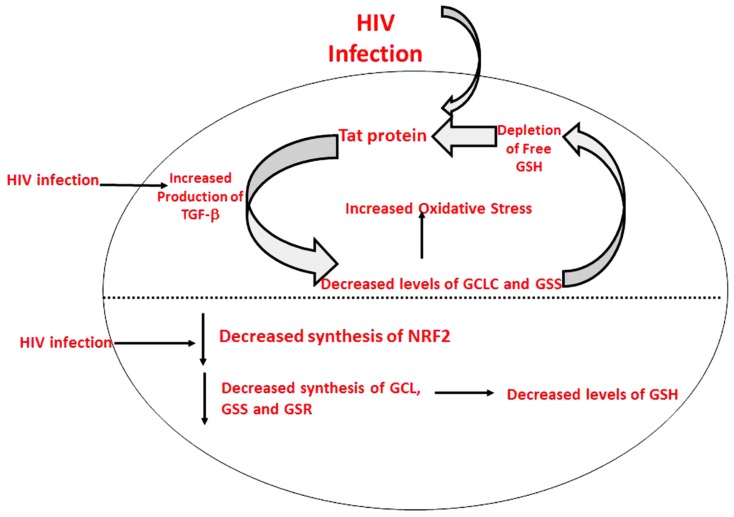
**Model illustrating causes for decreased levels of GSH in individuals with HIV infection**.

In this study we investigated the cause for decreased levels of GSH in individuals with HIV infection by quantifying the levels of GSS, GCLC, and GSR in the RBCs derived from healthy subjects and individuals with HIV infection. The results of the Western Blot indicate that there is a significant difference in the levels of GSS, GCLC, and GSR between HIV-infected individuals and healthy individuals, which supports our hypothesis that individuals with HIV infection have lower concentrations of enzymes that are responsible for both *de novo* synthesis of GSH and conversion of GSSG to GSH (**Figures 1–3**). In addition, our results indicate that there is a significant decrease in the levels of total GSH in the RBCs derived from HIV-infected individuals (**Figure 4**). Overall, these significant findings indicating lower levels of GSS, GCLC, and GSR in HIV-infected individuals support our hypothesis and contribute to previous findings that there are lower levels of GSH in HIV-infected individuals than healthy individuals (**Figure 5**). Observations from the current study combined with our previous findings strongly suggest that liposomal formulations of GSH can be used as a possible supplement to current HIV treatments since they can provide complete *r*GSH molecules, bypassing the cellular machinery for GSH production. Liposomal formulations containing GSH can be more effective in supplementing the intracellular *r*GSH and restoring the immune cell functions including the antimycobacterial activity in macrophages from HIV patients at concentrations lower than NAC (Venketaraman et al., 2006; Guerra et al., 2011, 2012; Morris et al., 2012, 2013).

## Conflict of Interest Statement

The authors declare that the research was conducted in the absence of any commercial or financial relationships that could be construed as a potential conflict of interest.
